# Top-Down Genomic Surveillance Approach To Investigate the Genomic Epidemiology and Antibiotic Resistance Patterns of Enterococcus faecium Detected in Cancer Patients in Arkansas

**DOI:** 10.1128/spectrum.04901-22

**Published:** 2023-03-30

**Authors:** Zulema Udaondo, Kaleb Abram, Atul Kothari, Se-Ran Jun

**Affiliations:** a Department of Biomedical Informatics, University of Arkansas for Medical Sciences, Little Rock, Arkansas, USA; University Paris-Saclay, AP-HP Hôpital Antoine Béclère, Service de Microbiologie, Institute for Integrative Biology of the Cell, CEA, CNRS

**Keywords:** *Enterococcus faecium*, population structure, genomic epidemiology, vancomycin, daptomycin, genomic surveillance, daptomycin resistance, vancomycin resistance

## Abstract

Control of hospital-associated Enterococcus faecium infection is a strenuous task due to the difficulty of identifying transmission routes and the persistence of this nosocomial pathogen despite the implementation of infection control measures that have been successful with other important nosocomial pathogens. This study provides a comprehensive analysis of over 100 E. faecium isolates collected from 66 cancer patients at the University of Arkansas for Medical Sciences (UAMS) between June 2018 and May 2019. In the top-down approach used in this study, we employed, in addition to the 106 E. faecium UAMS isolates, a filtered set of 2,167 E. faecium strains from the GenBank database to assess the current population structure of E. faecium species and, consequently, to identify the lineages associated with our clinical isolates. We then evaluated the antibiotic resistance and virulence profiles of hospital-associated strains from the species pool, focusing on antibiotics of last resort, to establish an updated classification of high-risk and multidrug-resistant nosocomial clones. Further investigation of the clinical isolates collected from UAMS patients using whole-genome sequencing analytical methodologies (core genome multilocus sequence typing [cgMLST], core single nucleotide polymorphism [coreSNP] analysis, and phylogenomics), with the addition of patient epidemiological data, revealed a polyclonal outbreak of three sequence types occurring simultaneously in different patient wards. The integration of genomic and epidemiological data collected from the patients increased our understanding of the relationships and transmission dynamics of the E. faecium isolates. Our study provides new insights into genomic surveillance of E. faecium to assist in monitoring and further limiting the spread of multidrug-resistant E. faecium.

**IMPORTANCE** Enterococcus faecium is a member of the gastrointestinal microbiota. Although its virulence is low in healthy, immunocompetent individuals, E. faecium has become the third leading cause of health care-associated infections in the United States. This study provides a comprehensive analysis of over 100 E. faecium isolates collected from cancer patients at the University of Arkansas for Medical Sciences (UAMS). We employed a top-down analytical approach (from population genomics to molecular biology) to classify our clinical isolates into their genetic lineages and thoroughly evaluate their antibiotic resistance and virulence profiles. The addition of patient epidemiological data to the whole-genome sequencing analytical methodologies performed in the study allowed us to increase our understanding of the relationships and transmission dynamics of the E. faecium isolates. This study provides new insights into genomic surveillance of E. faecium to help monitor and further limit the spread of multidrug-resistant E. faecium.

## INTRODUCTION

Enterococci are common members of the microbiota of the gastrointestinal tract and other mucocutaneous membranes (1, 2). Although their virulence is low in healthy, immunocompetent individuals, enterococci can cause serious infection in patients in health care settings, with an estimated 5,400 deaths in 2017 (3). It has been reported that Enterococcus is one of the genera that most readily colonize the intestine after depletion of the indigenous intestinal microbiota, which occurs following therapeutic treatments, such as the use of antibiotics (4). More importantly, the development of Enterococcus faecium carriage in the gut has been highlighted as the most important risk factor for E. faecium bacteremia in immunocompromised patients (5, 6).

In the United States, recent reports indicate that 75% to 80% of the E. faecium infections reported in nosocomial settings are caused by vancomycin-resistant Enterococcus faecium (VREfm) (7). VREfm strains that cause these infections in hospital settings are commonly resistant to all antienterococcal antibiotics (such as ampicillin and aminoglycosides) that have been widely described (8, 9), making these infections often untreatable (10, 11). For that reason, VREfm is currently on the World Health Organization’s (WHO) list of high-priority pathogens for research and development of new antibiotics (12).

Daptomycin (DAP) is a cyclic lipopeptide antibiotic that has become the cornerstone in the therapeutic treatment for severe infections caused by VREfm and methicillin-resistant Staphylococcus aureus (MRSA) infections (13–15). As a last resort antibiotic, DAP is used in combination with other drugs or in sequential regimens after the failure of standard treatments (16) such as vancomycin (VAN) for infections caused by VREfm (17). Although DAP resistance in E. faecium is still rare, there has been an increase in recent years of treatment failures related to an emergence of DAP-nonsusceptible strains (18, 19). Major institutions and health care centers have reported that 20% to 30% of their VREfm isolates are DAP-nonsusceptible (DAP-NS) (20). Current knowledge suggests that the DAP-NS phenotype is the result of a complex, multifactorial mechanism which has its origin in specific mutations in the chromosome of the resistant strains (14). These mutations have been most consistently observed in two genes (liaR and liaS) that encode proteins related to phospholipid metabolism and cell envelope homeostasis (11, 21).

Whole-genome sequencing (WGS) methods have become crucial for outbreak and surveillance investigations in different settings due to their high resolution and backward compatibility with traditional methods, such as multilocus sequence typing (MLST) (5, 22–25). These techniques have been successfully leveraged to investigate the transmission of E. faecium strains between livestock and humans (26), between multiple hospitals (27), and in hospital settings (19, 28). Thus, surveillance methods for infection prevention and control are rapidly moving toward integration of genomic and epidemiology data. The use of WGS-based surveillance of VREfm combined with epidemiological information is particularly relevant, as there are a great number of epidemiologically unrelated cases that are caused by asymptomatic colonization, hampering the detection of smaller outbreaks (23) and acting as a reservoir for onward transmission (24).

In this study, we applied a top-down (from population genomics to molecular biology) genomic surveillance approach to investigate the genomic epidemiology and the antibiotic resistance patterns of nosocomial E. faecium strains, especially focusing on their VAN and DAP resistance genotypes. For this purpose, we employed all publicly available E. faecium genomes in GenBank (at the time of the study) and a set of 106 E. faecium isolates from 66 cancer patients collected between June 2018 and May 2019 at the University of Arkansas for Medical Sciences (UAMS) (Arkansas, USA). The goal of the present study was 2-fold. The first aim was to leverage WGS and comparative genomics to expand the current knowledge of E. faecium distribution in its three clades (A1 [hospital-associated isolates], A2 [environmental and animal-associated isolates and human commensals that could cause occasional human infections], and B [community-associated isolates]) in order to analyze the antibiotic resistance patterns of hospital-associated E. faecium strains. The second aim was to describe the clinical and epidemiological traits of 106 clinical E. faecium strains isolated from UAMS cancer patients to better understand E. faecium epidemiology and transmission routes in the hospital setting.

## RESULTS

### The genomic population structure of Enterococcus faecium species produces a seamless differentiation of three genetic lineages.

**FIG 1:**
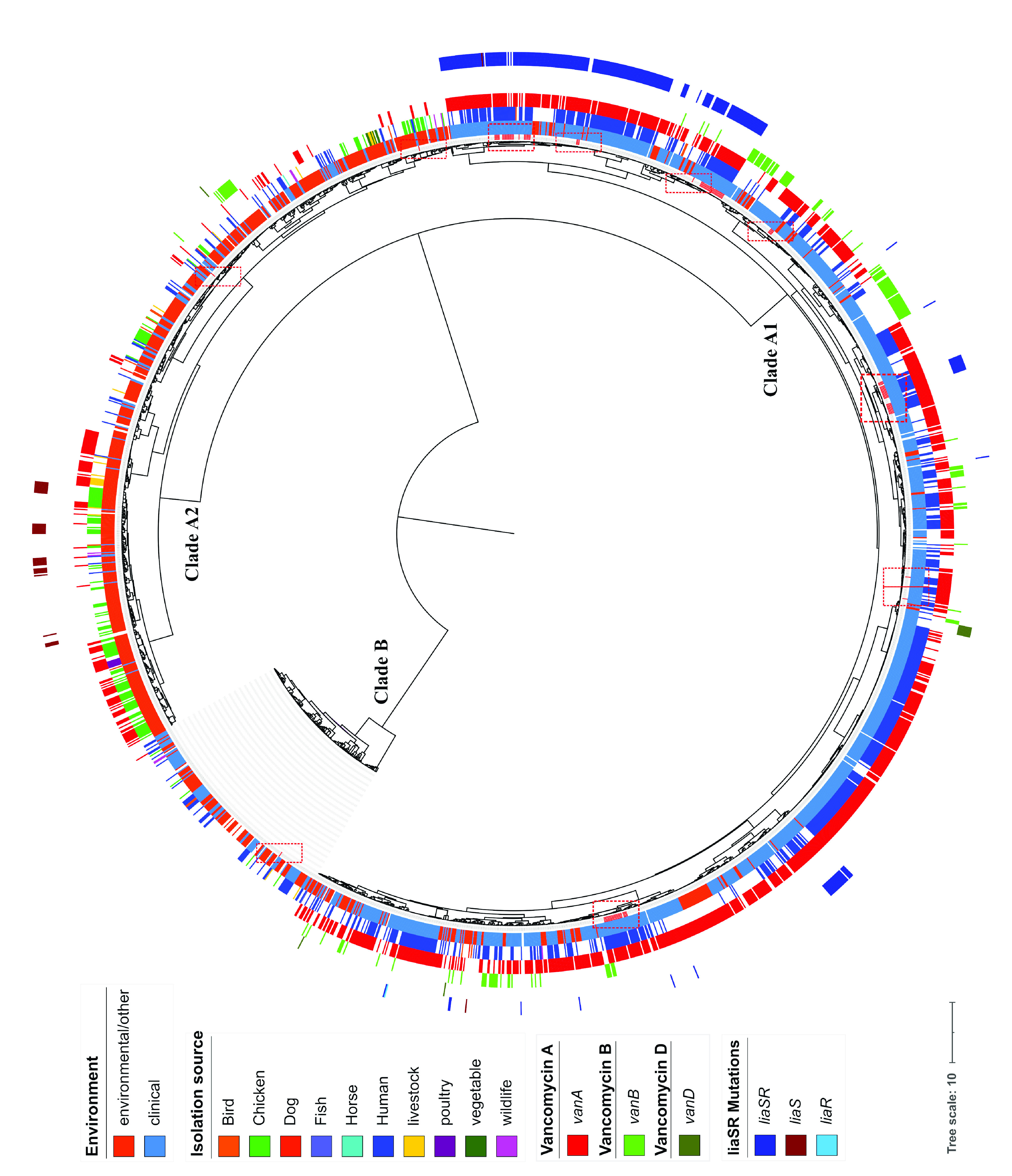
Pairwise genomic distance-based tree. Pairwise genomic distances were obtained using FastANI. The tree shows a clear division of the species into clades A1, A2, and B. The first ring indicates whether the isolate was environmental or clinical. The second ring represents the isolation source. The presence of the vancomycin resistance determinants VanA, VanB, and VanD is represented in rings 3 to 5. The outer ring represents the presence of mutations in *liaSR* genes, related to development of the DAP-nonsusceptible phenotype. The location on the tree of the 106 E. faecium isolates collected for this study is indicated with dashed red boxes.

The genome distances tree (Fig. 1) of the entire set of genomes of E. faecium strains available to date (June 2021) in the GenBank database (*n* = 2,167 E. faecium strains) and the set of UAMS isolates (*n* = 106 E. faecium strains), constructed using Fast alignment-free computation of whole-genome average nucleotide identity (FastANI) (29), shows a clear division of the strains of this species into the two previously described groups, A and B, as well as a subsequent division of the A strains into clades A1 and A2 (see Table S1 in the supplemental material) (30, 31). Clade A1 (1,384 total strains), formerly known as clonal complex 17 [CC17]) (32, 33), harbors strains obtained almost entirely from clinical and human sources (Fig. 1, blue inner ring), with only one “avian” source sample, while clade A2 (*n* = 695 strains) comprises E. faecium strains mainly from environmental and animal sources (Fig. 1, orange inner ring). Clade B (n = 194 strains) consists of strains that were isolated from environmental sources (mainly chickens and cattle). However, this clade also harbors many strains classified as clinical strains from human samples (30) that are usually found in the gut microbiome of healthy individuals and rarely cause infections (34).

According to the classification obtained in our analysis, the set of 106 E. faecium strains isolated from UAMS patients have representatives in all three clades, with most of them (a total of 103 isolates) classified in clade A1. However, two isolates from two patients (UAMS_EF26 and UAMS_EF79) were classified in clade A2 and one in clade B (UAMS_EF24).

Isolate UAMS_EF26 was identified in the urine sample of a cancer patient at the emergency department and is genetically highly similar to E. faecium strain 306EA1 (ANI, 99.9003), which was isolated from the breast meat of a chicken processed in the state of Texas (USA) according to a study of enterococci associated with chicken meat from U.S. supermarkets (35). Strain 306EA1 was also classified as clade A2 in the study performed by Manson and colleagues (35). Isolate UAMS_EF79, also obtained from the urine sample of a patient with cirrhosis in urinary analysis, was genetically closely related to E. faecium strain B3754 (ANI, 99.8334), which was isolated from the stool sample of a human neonate in the study by Liang and colleagues (36).

As previously stated, one isolate from the set of UAMS samples (UAMS_EF24), isolated from an outpatient in a routine urine analysis, was identified as belonging to clade B. Isolate UAMS_EF24 is closely related to E. faecium Com15 (ANI, 99.5602), which was obtained from the stool of a healthy individual (37) and classified as clade B in several studies (38, 39).

Our ANI-based lineage classification was further validated using the data set provided by Lebreton et al. in 2013 (30), which identified the three E. faecium clades (A1, A2, and B) using a single nucleotide polymorphism (SNP)-based phylogenetic tree. We found only one discrepancy in the lineage classification between data sets, E. faecium strain E4452, which was classified as clade A1 by the SNP-based lineage classification but clade A2 based on our ANI-based methodology. E. faecium strain E4452 is an ampicillin-resistant canine isolate assigned to ST266, a sequence type commonly found in dogs (40), thus fitting better with the characteristics of clade A2 (animal-associated isolates).

Due to the growing number of VREfm infections in hospital settings, which now represent up to 80% of the E. faecium infections in some areas of the United States (7, 9), we studied the distribution of VAN resistance determinants in the context of the species, by mapping strains for which these determinants were found on the genome distance tree in Fig. 1 (third to fifth rings of the tree). This analysis revealed that clade A1 harbored the highest number of VAN-resistant strains with *vanA* (1,053 clade A1 strains out of 1,193 total strains with the *vanA* operon identified in the species set), *vanB* (143 clade A1 strains out of 160 total strains with the *vanB* operon identified in the species set), and *vanD* (8 clade A1 strains out of 10 total strains with the *vanD* operon identified in the species set) genotypes (note that VanC-type resistance is ordinarily intrinsic to Enterococcus gallinarum and Enterococcus casseliflavus) (41).

It has been reported that the use of VAN analogues such as avoparcin as a growth promoter on poultry, pig, and cattle farms in Australia and the European Union (EU) promoted the proliferation of VAN resistance in farm settings (42–44). Our analysis shows that despite the ban of several antibiotics used as growth promoters, including avoparcin, which has been banned since 1997 in the EU, over 22% of the strains from clade A2 still harbor antibiotic resistance determinants for the VAN resistance genotype *vanA* (*n* = 139), *vanB* (*n* = 17), or *vanD* (*n* = 2) (note that the oldest strain used in our data set was released in GenBank in 2002 and the newest in 2021) (Table S1).

As reported in several studies, LiaR (W73C) and LiaS (T120A) amino acid substitutions in proteins encoded in the LiaFSR (for lipid II-interacting antibiotics) three-component cell envelope stress response system, which regulates cell envelope integrity, are associated with the development of a DAP-nonsusceptible phenotype in E. faecium (19, 21, 45). We queried our species set and identified 275 E. faecium strains (12% of the total species set) that harbored those specific comutations in *liaSR* genes (Fig. 1, outer ring). These strains were classified as clade A1, and a high proportion of them (243/275 [88%]) also carried VAN resistance determinants. However, in the set of UAMS isolates, 63 of 106 isolates (59%) had mutations related to the DAP-nonsusceptible phenotype in *liaSR* genes, and 59 of them (94%) also carried the *vanA* gene cluster, thereby implying that the number of isolates with mutations related to the DAP-nonsusceptible phenotype is considerably overrepresented in the set of UAMS isolates.

### Resistome and multilocus sequence typing analysis of clade A1 strains indicate predominance of multidrug-resistant clone ST80.

**FIG 2:**
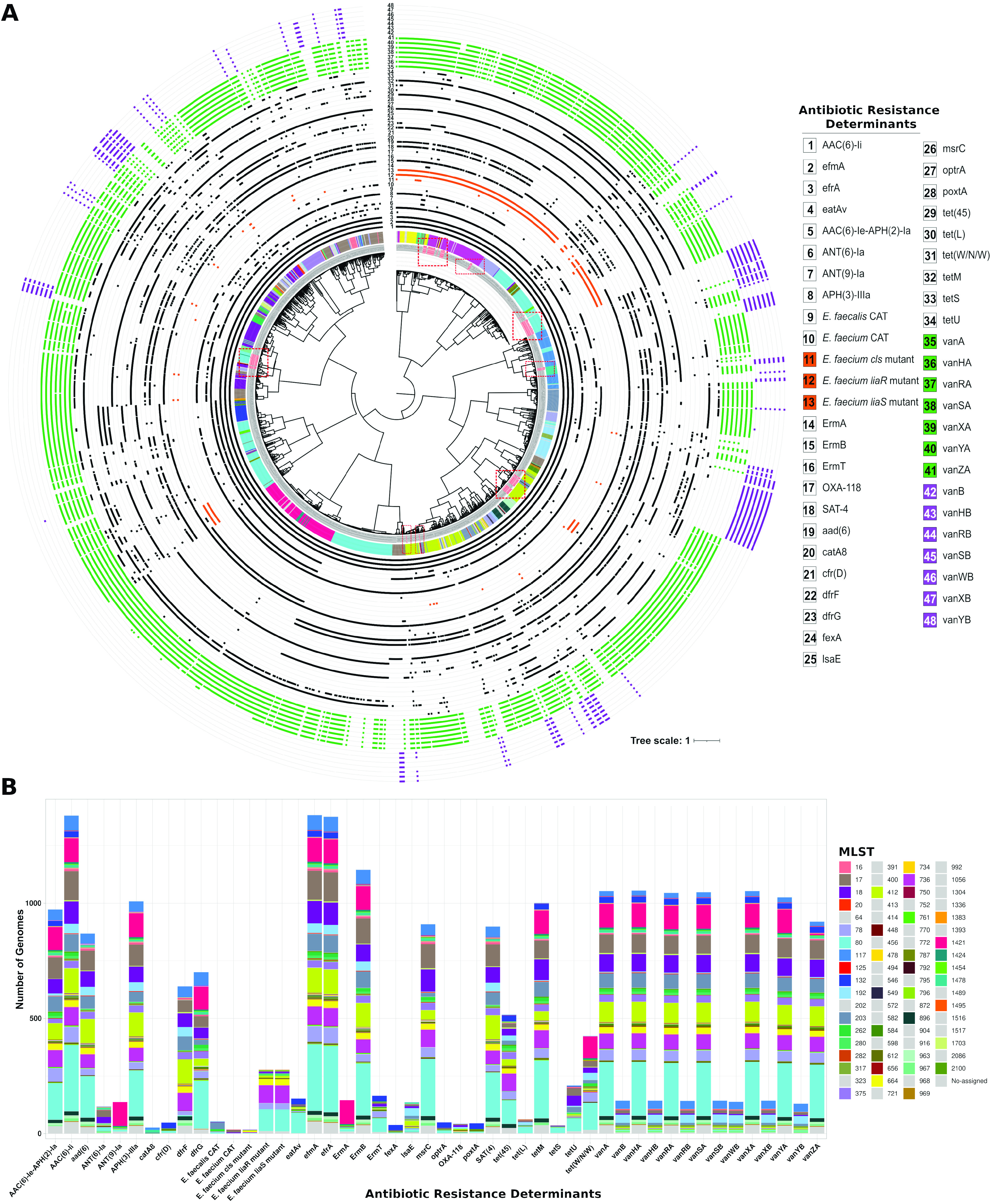
(A) Pairwise genomic distance-based tree using ANI measurements of 1,384 clade A1 E. faecium strains. The outer rings of the tree represent the resistome of each strain. The innermost ring represents sequence type affiliation. The location on the tree of the 106 E. faecium isolates collected for this study is highlighted with dashed red boxes. (B) Stacked bar plot of ARDs identified in strains from clade A1 with MLST distribution. A total of 48 nonredundant ARDs and 75 sequence types were annotated for strains in clade A1.

The hospital-adapted lineage clade A1, which accounts for the vast majority of E. faecium invasive infections (5, 46), harbors the majority of VREfm strains, including most of the UAMS isolates. To further characterize the resistome of clade A1 strains, we identified the antibiotic resistance determinants (ARDs) of the 1,384 strains belonging to clade A1 using the Resistance Gene Identifier (RGI) tool against the Comprehensive Antibiotic Resistance Database (CARD) (47). A total of 48 ARDs (found in more than 1% of clade A1 strains) were annotated (Fig. 2 and Table S2). Moreover, using *in silico* MLST, we classified a total of 1,332 clade A1 strains into 74 sequence types (STs) that were interspersed throughout the tree shown in Fig. 2A (inner ring).

The *in silico* MLST analysis revealed that the most predominant STs in clade A1 are ST80 (287 strains), followed by ST17 (126 strains), ST412 (107 strains), ST1421 (104 strains), and ST18 (97 strains), which together account for more than 54% of clade A1 strains. Four of these STs were also among the STs found to have a higher level of multidrug resistance (in order of multidrug resistance level: ST17, ST80, ST18, and ST412). In the case of ST17, all 48 antibiotic resistance determinants were found in the resistome of strains belonging to this sequence type (Table S2). However, two ARDs were not found among ST80 strains. Those two ARDs were chloramphenicol acetyltransferases (CAT) encoded by *catA8* and the Enterococcus faecalis CAT. In the case of ST18 strains, two ARDs, the rRNA-methylating enzyme encoded by *cfr*(D), which confers resistance to several classes of antimicrobial agents, such as lincosamides, phenicols, and oxazolidinones, and the ATP-binding cassette protein that confers resistance to tetracyclines, phenicols, and oxazolidinones, encoded by *poxt*(A), were not found in the resistome of the strains from this ST.

Notably, 12 of 74 STs do not harbor strains with *vanA* or *vanB* ARDs (these were ST400, ST202, ST456, ST598, ST64, ST323, ST582, ST549, ST752, ST904, ST916, and ST413). These 12 STs are among the least representative STs in clade A1 strains, having only one classified strain (two in the case of ST549) and being among the STs with the lowest number of annotated ARDs. Dominant STs with the largest proportion of VREfm are ST1421 (99% of strains in this sequence type are VREfm), ST203 (85% of strains are VREfm), ST736 (99% of strains are VREfm), and ST80 (81% of strains are VREfm).

Clade A1 strains are usually resistant to ampicillin and quinolone and have a higher rate of spontaneous mutation and recombination than clades A2 and B (33, 48). The stacked bar plot in Fig. 2B shows that the most abundant ARDs in clade A1 strains are the intrinsic *efmA* (which is present in 99.9% of clade A1 strains and confers resistance to macrolides and fluoroquinolones) and the chromosomal *aac(6′)-Ii* gene, which was found in 99.8% of strains and confers resistance to aminoglycosides. The *efrA* gene encodes the EfrA efflux pump subunit and confers resistance to several antimicrobials, such as rifamycin, fluoroquinolone, and macrolides. This ARD was reported for the first time in E. faecium species in 2013 (49) and was annotated in 99.4% of clade A1 strains. The acquired ARD e*rmB*, which is induced by erythromycin and confers resistance to macrolides, lincosamides, and streptogramin B (known as the MLS_B_ phenotype), was annotated in 83% of clade A1 strains, followed by the *vanA* ARD, whose gene cluster was annotated in 76% of clade A1 strains.

The distribution of STs shown in the stacked bar plot also indicates that there are some ARDs that can be found in virtually all STs (mainly those described above), but on the other hand, there are some ARDs that are found in certain STs and not in others. For example, the chromosomal dihydrofolate reductase gene, *dfrF*, which confers resistance to diaminopyrimidine, was annotated in almost 50% of clade A1 strains and is predominantly found in strains belonging to clones ST412 and ST736 (99% of strains belonging to these two STs harbor *dfrF*). In contrast, this ARD was found in only 1% of strains classified as ST80 and in 3% of ST736 strains. On the other hand, the dihydrofolate reductase gene, *dfrG*, was annotated in 72% of ST80 strains and 95% of ST1421 strains but in only in a small percentage of ST412 and ST736 strains (29% and 2%, respectively) (Table S2). In the same vein, strains with mutations in genes encoding the LiaFSR three-component regulatory cell envelope stress response system, associated with DAP nonsusceptibility (21), were found in 100% of the strains from sequence types ST736, ST1454, and ST664. These comutations were also found in 67% of strains classified as ST280 and in 33% of strains classified as ST80 and ST734. Only a small proportion of clade A1 strains (1%), mainly classified as ST17, had mutations in the cardiolipin synthase (*cls*) gene, which has also been associated with a predisposition to nonsusceptibility to DAP (50).

Along with DAP, linezolid has become a last-resort antibiotic for the treatment of VREfm infections since its introduction for clinical use in 2000 in the United States (51). In recent years, the increased use of linezolid has led to an extensive growth in the number of linezolid-resistant VREfm infections (52). Enterococcal linezolid resistance can be due to chromosomal mutations in the 23S rRNA gene and to mutations in the genes encoding L3 and L4 ribosomal proteins (53). However, resistance can also be developed after the acquisition of the *optrA*, *poxtA*, and *cfr*-like ARDs (53, 54). *optrA*, *poxtA*, and *cfrD* were annotated in 49, 45, and 48 clade A1 strains, respectively. The distributions of these three ARDs among the STs are very similar (Fig. 2), and strains with these three ARDs primarily belonged to ST132. Thus, 92% of strains from ST132 harbor the 23S rRNA methyltransferase gene *cfrD*, and more than 88% and 96%, respectively, also harbor the *optrA* and the *poxtA* genes, which encode two ribosomal protection proteins of the ABC-F protein family.

*In silico* virulence profiling of clade A1 strains using ABRicate v1.0.0 (T. Seemann [GitHub]; https://github.com/tseemann/abricate) and a virulence factor custom database (see Materials and Methods) unveiled a total of 4,735 virulence factors in clade A1 strains codified by 12 genes (*ace*, *acm*, *ecbA*, *esp*, *gspG*, *hitB*, *hyl*, *ipxA*, *msbB*, *scm*, *sgrA*, and *wecA*). The most predominant virulence factors found are the LPxTG-type surface-exposed anchored proteins, such as the collagen-binding microbial surface component Acm, which recognizes adhesive matrix molecules (MSCRAMMs) and was annotated in 91% of clade A1 strains, and the LPxTG surface adhesin SgrA, which was found in 86% of clade A1 strains. The SgrA virulence factor binds to fibrinogen and nidogen and is commonly implicated in biofilm formation. The sequence types with the highest number of virulence factors were ST80, with a total of 916 annotated virulence factors (Table S3), ST1421, with a total of 439 virulence factors, and ST17 and ST18, with 427 and 320 annotated virulence factors, respectively. Nevertheless, the profile of virulence factor abundance varies among the four STs (Fig. S1). For example, although members of ST1421 were not among the most highly multidrug-resistant strains of the analysis, this sequence type has the highest percentage of strains harboring most of the virulence factors annotated in clade A1 strains. Thus, 100% of the ST1421 clade A1 strains harbored genes for the virulence factors Acm (nonfimbrial adhesin) and EcbA (nonfimbrial adhesin) in their genome; 99% of ST1421 strains had the virulence factor SgrA (nonfimbrial adhesin), and 98% harbored the virulence factor Hyl (hyaluronidase) (Table S3).

### Genomic epidemiology of 106 E. faecium isolates from cancer patients in Arkansas reveals persistence of clones in urine samples.

In this study, we retrospectively sequenced the whole genomes of 106 E. faecium isolates obtained from 66 cancer patients ranging in age from 23 to 87 years. All patients presented underlying medical conditions, and 17 of them had more than one sample positive for E. faecium, with a range of 2 to 12 isolates per patient (Table S4). The 106 isolates were classified in 13 STs, of which the most predominant were ST80 (*n* = 39), ST412 (*n* = 20), and ST736 (*n* = 20). A total of 34 ARDs were identified in the set of 106 isolates (Table S5; Fig. S2). More than 90% of the isolates possessed ARDs for vancomycin (*vanA*), tetracycline (*tetM*), erythromycin, macrolide and streptogramin B antibiotics (*msrC*), and fluoroquinolones (*efmA*). The ARDs AAC(6′)-Ii and *efrA*, conferring resistance to aminoglycosides, macrolides, and fluoroquinolones, were found in all 106 isolates collected in this study. Although 60% of the isolates harbored mutations in *liaSR* genes related to development of nonsusceptibility to daptomycin, linezolid ARDs were not found among the 106 isolates obtained for this study. Analysis of the virulence factors annotated in the set of isolates showed that the nonfimbrial adhesin (Acm) was the most prevalent virulence factor, as it was found in all 106 isolates collected. Other virulence factors identified among these isolates were the nonfimbrial adhesins EcbA and SgrA, the fibrinogen-binding Fss3, and the collagen-binding Scm (Table S6). No other virulence factors such as gelatinase, hemolysin, or the enterococcal surface protein Esp (55), were detected in the set of isolates.

**FIG 3:**
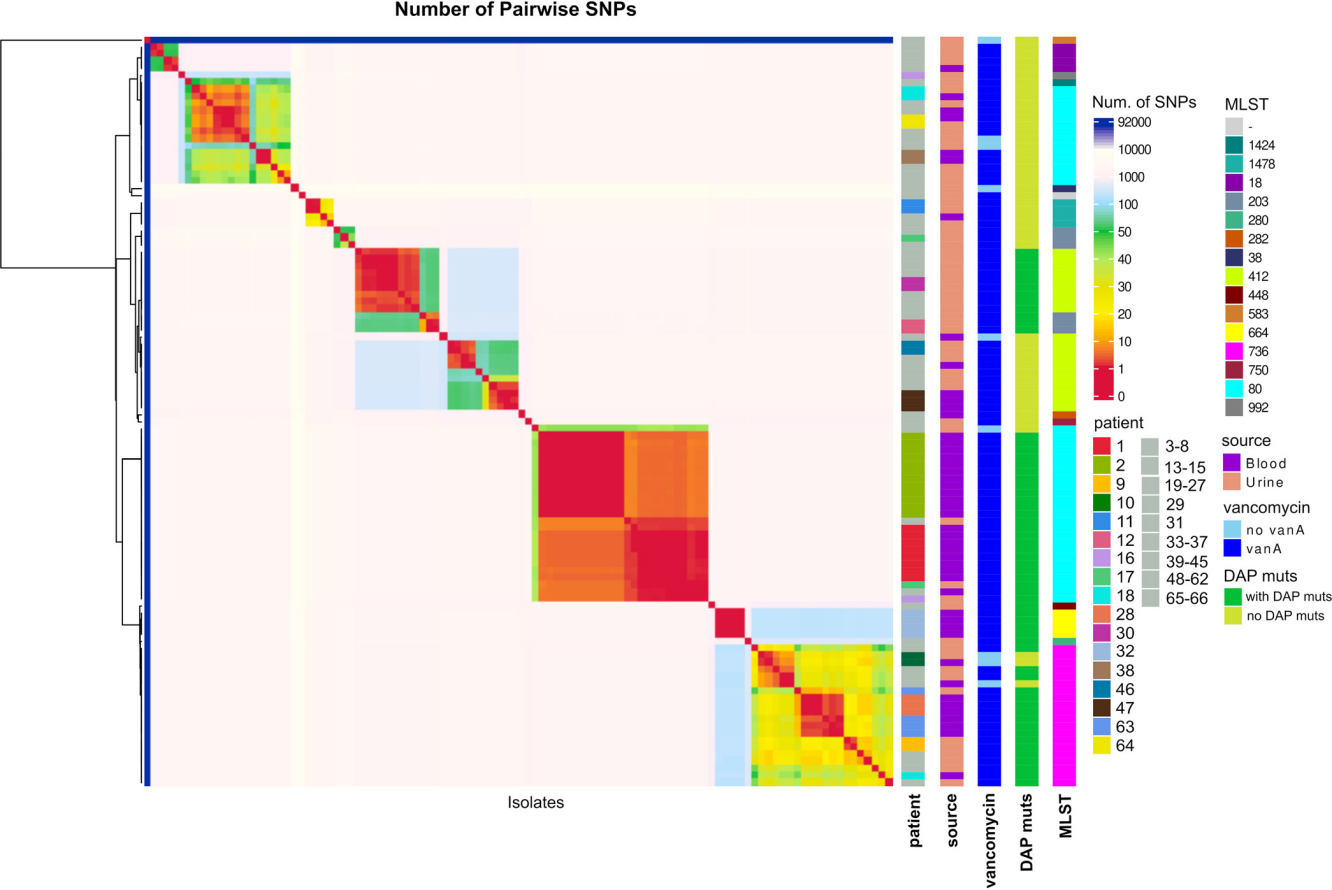
Pairwise coreSNP heat map of 106 E. faecium isolates from 66 cancer patients. The heat map was colored using pairwise coreSNP distance. Warmer colors in the heat map represent clusters of E. faecium clones. Metadata, including patient number, sequence type, isolation source, presence of *vanA* cluster, and presence of daptomycin nonsusceptibility related mutations, are annotated. Data for patients with only one isolate are shown in gray.

**FIG 4:**
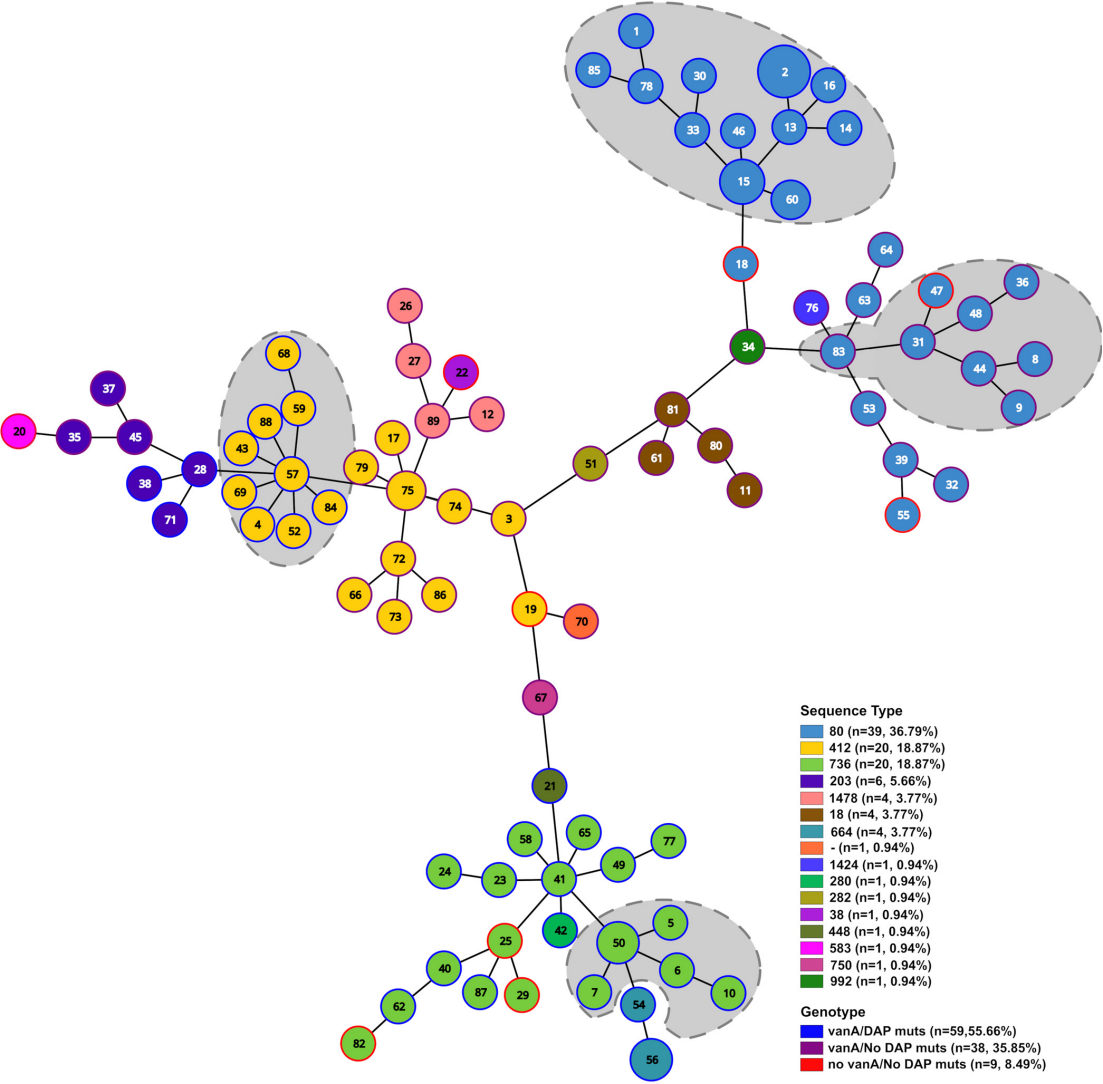
Minimum spanning tree of 106 E. faecium isolates from cancer patients. Numbers in nodes represent the cgMLST classification. Node size represents the number of strains for each cgMLST. Node color represents the sequence type. Different combinations of presence/absence of the *vanA* cluster and DAP nonsusceptibility-related mutations are shown in the outer ring of each node in blue, purple, and red. Percentages in parentheses are the relative abundances of strains in that category. A gray background delineates isolates that were detected as part of a clonal cluster with a coreSNP threshold of 15.

SNP-based methods are widely used for hospital outbreak investigation, as they retain higher resolution for tracking transmissions between patients (56). Thus, we constructed a pairwise coreSNP distance matrix with the set of UAMS isolates. The coreSNP distance matrix was plotted as a clustered heat map that shows several clusters of highly genetically related isolates differing by fewer than 50 SNPs (Fig. 3). The isolates within these clusters belong to the same sequence type and are, in almost all cases, independent of isolation source (blood or urine). The clusters with fewer than 50 SNPs were also confirmed by core genome MLST (cgMLST) analysis using the official E. faecium scheme with 1,423 core genes (57). The number of cgMLST allelic differences between all pairs of isolates was calculated and compared with the coreSNP distance matrix. Isolates with at least 5 allelic differences were classified into different complex types (CTs), yielding a total of 89 CTs for all 106 isolates (Fig. 4).

Based on previous VREfm surveillance studies, patients with isolates differing by fewer than 15 SNPs are considered closely related genetically and within the limits of putative transmission (23, 58). Using the threshold of 15 pairwise coreSNPs, we identified four major E. faecium clonal clusters prevalent among UAMS samples that were collected at different time points over 1 year (Tables S4 and S7). Isolates belonging to these four clonal clusters had 14 or fewer cgMLST allelic differences. Manual inspection of the coreSNPs found between strains belonging to the four clonal clusters showed a limited number of recombination events (0 to 2 in the set of strains) that were not taken into account in our threshold.

**FIG 5:**
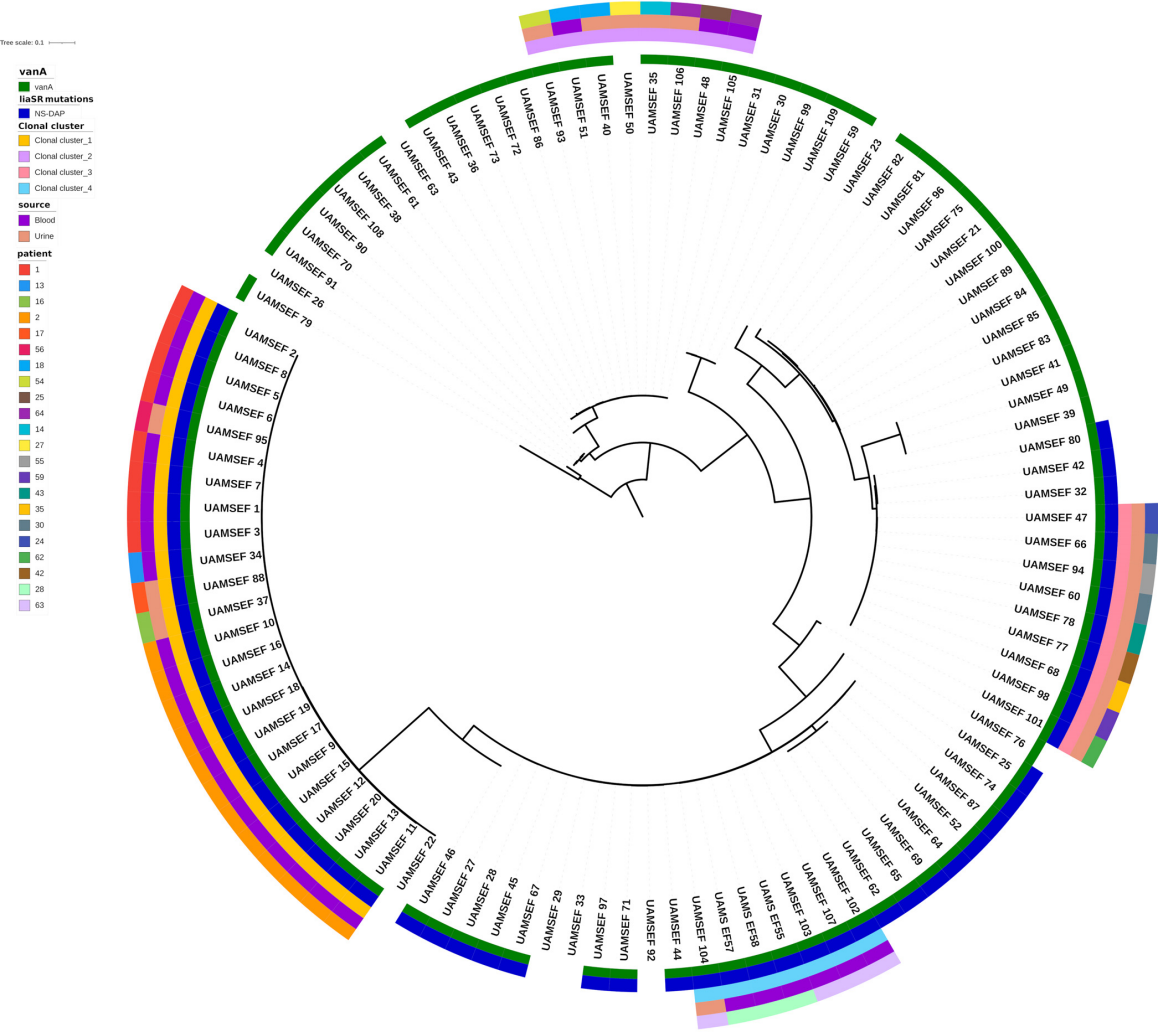
Maximum-likelihood phylogenetic tree of 105 clade A1 and A2 E. faecium isolates. The phylogeny of the 105 isolates was obtained using the alignment of 1,266 core genome alleles and IQ-TREE software with a maximum-likelihood approach. Isolates belonging to each of the four clonal clusters are marked in the inner ring of the tree. The presence of the *vanA* cluster of genes conferring resistance to vancomycin and mutations in *liaSR* genes related to the daptomycin-nonsusceptible phenotype are represented in rings 2 and 3, respectively.

The four major clonal clusters were further investigated using a maximum-likelihood tree of 103 clade A1 isolates which was rooted with the two UAMS isolates (UAMSEF_26 and UAMSEF_79) placed in clade A2 (Fig. 5; Fig. S3 to S7). Analysis of the resistance profile of the isolates belonging to each of the clonal clusters showed distinctive antibiotic resistance profiles for each of the clonal clusters (Fig. S2). The main epidemiological characteristics of the four main clonal clusters of this polyclonal outbreak are described below.

**(i) Clonal cluster 1 of 24 VREfm ST80 isolates with DAP-nonsusceptible mutations.** Clonal cluster 1 involved isolates that were obtained from six different patients with different underlying hematologic malignancies (Table S7). Four of these inpatients shared the same bed unit at different time points. At the time of isolate collection, patient 17 was an outpatient of the UAMS cancer infusion center with a history of prior admissions to the hospital, with dates overlapping those for other patients at the time of higher prevalence of this clone at the hospital, in which the same clone was found in at least two other hospitalized patients concurrently (Tables S3 and S7). Most of the isolates from this cluster belong to two patients (isolates UAMSEF_01 to UAMSEF_08 from patient 1 and UAMSEF_09 to UAMSEF_20 from patient 2) whose specific cases were previously described by us (19) due to the observed development of the DAP-nonsusceptible phenotype during treatment with DAP of these two patients. According to the phylogenetic analysis (Fig. 5 and Fig. S3 and S4), isolates UAMSEF_34, UAMSEF_37, and UAMSEF_88 originated from the lineage of the isolate from patient 1. Remarkably, isolates UAMSEF_88 and UAMSEF_95 were both isolated from urine samples from two different patients, collected 3 and 4 months after the discharge of the last patient registered in the hospital and from whom strains belonging to this cluster were isolated. The integration of genomic and epidemiologic data indicates an extended prevalence of this clone in the gastrointestinal or urinary tract of the patients several months after the time of infection.

**(ii) Clonal cluster 2 of eight ST80 isolates with diverse vancomycin genotypes.** Clonal cluster 2 comprises isolates found in six patients who had different underlying conditions, including carcinoma, cholangiocarcinoma, and acute lymphoblastic leukemia. Most of the patients from this cluster had overlapping hospital stays (Table S7). However, all patients were hosted in different bed units, indicating that E. faecium clones may have spread through different wards. None of the isolates from this cluster harbored DAP nonsusceptibility-related mutations in *liaSR* genes, and one isolate (UAMSEF_50) even showed a vancomycin-susceptible phenotype (the *vanA* cluster was also not found in this isolate). According to cgMLST, coreSNP, ANI, and phylogenetic analyses, isolate UAMSEF_50 is most closely related to UAMSEF_35. As in clonal cluster 1, we observed the presence of isolates from urine samples which harbored very high genetic similarity to isolates collected from patients who had been discharged several months earlier (UAMSEF_93 and UAMSEF_106), indicating a possible long-term prevalence of this clone in outpatients.

**(iii) Clonal cluster 3 of nine ST412 VREfm and with DAP-nonsusceptible mutations.** The clonal cluster 3 outbreak involved isolates obtained from urine samples from eight different patients who had different underlying conditions, mainly related to hematological malignancies. Patients remained in different bed units in most cases. Interestingly, although all isolates in this outbreak have the *vanA* gene cluster, responsible for the vancomycin-resistant phenotype, one of the isolates (UAMS_EF47 from patient 24) had a vancomycin-susceptible phenotype. This isolate was collected at the emergency department during the first day of admission of patient 24. Since this patient was not hospitalized at the time of sample collection, the clone identified in the urine sample was designated community acquired. However, examination of the epidemiological data revealed that patient 24 had been previously admitted to the hospital due to pancreas and kidney transplantation and was on prophylactic antibiotic treatment, indicating a possible long-term prevalence of this clone in this patient.

### (iv) Clonal cluster 4 of six ST736 VREfm isolates with DAP-nonsusceptible mutations.

Isolates from two patients diagnosed with acute myeloid leukemia and end-stage renal disease make up clonal cluster 4. Both patients remained in different bed units, and their discharge and admission occurred on consecutive days.

## DISCUSSION

In order to understand the distribution of a large collection of E. faecium clinical isolates collected from cancer patients at UAMS, we first leveraged all the genomes available in the GenBank database (as of June 2021) and constructed the genomic population structure of E. faecium species using pairwise ANI distances. The subdivision of E. faecium strains into clades A1 and A2 has been suggested in previous studies (46). However, the use of a larger data set with strains from broader geographical and ecological origins in combination with WGS-based methodologies illustrated a strikingly clear distribution of the three clades of E. faecium species (Fig. 1). Clade A1 strains dominate the hospital population of disease-causing E. faecium (59, 60). Thus, most of the isolates obtained in our surveillance study on cancer patients at high risk were classified as members of E. faecium clade A1 (60).

Although clade A2 strains were isolated mainly from animal sources, we identified 54 strains in clade A2 obtained from human samples. Most A2 strains from humans, as in the case of strain UAMS_EF26 from the set of UAMS samples, have a high sequence similarity to strains obtained from animal sources. Evidence of transient intestinal colonization by E. faecium strains of animal origin, capable of transferring mobile elements with resistance genes to the host microbiota, has been provided in other studies (61–63), imposing a potential threat to patients who have weakened immunity and whose gut microbiome is under selective pressure from antibiotics.

We then focused our analysis on the clade A1 strains. The *in silico* MLST classification of clade A1 strains resulted in 74 STs. The ability of MLST analysis to discern between E. faecium strains has been discussed in multiple studies, which reached different conclusions due to the high frequency of recombination events in this species that can affect several of the genes employed in MLST analysis (46). However, due to the rapid and unambiguous procedure, MLST is still widely used for global and long-term epidemiology of E. faecium and other bacterial species (64, 65). In line with previous studies, the current E. faecium MLST scheme does not accurately reflect the population structure of the clade A1 E. faecium strains (Fig. 2A) (66). However, it seems to maintain a certain degree of correspondence with several monophyletic groups observed in the genomic distance tree of clade A1 strains (Fig. 2A). Therefore, for genomic surveillance purposes, early identification of E. faecium isolates by their STs may still be useful as an initial preventive infection control measure, especially if there is prior knowledge of the resulting sequence type as belonging to a multidrug-resistant and pathogenic lineage (59).

Due to the above observations, we further investigated the possible relationships between the antibiotic resistance determinants, virulence factors, and the sequence types of clade A1 strains. Our results showed that currently, the most predominant sequence type in clade A1 strains is ST80 (287 strains), followed by ST17 (126 strains), ST412 (107 strains), ST1421 (104 strains), and ST18 (97 strains), which together account for more than 54% of clade A1 strains. Among these sequence types were the STs with the highest level of multidrug resistance (ST17, ST80, ST18, and ST412, in order of multidrug resistance level). ST1421 had the highest percentage of strains harboring most of the different classes of virulence factors annotated in clade A1 strains. This information follows the trend already observed in E. faecium of the emergence of highly hospital-adapted clones with increased development of multidrug resistance, which is related to the broad ability of E. faecium to survive in hostile antimicrobial-rich environments (67, 68). The process by which multidrug-resistant clones develop is known as “genetic capitalism” (69), a term used to reflect the fact that an increase in fitness through the accumulation of adaptive elements, such as ARDs, by specific clones in selective environments increases the probability of acquiring more adaptive elements, leading to the emergence of high-risk multidrug-resistant clones, as in the case of clade A strains (53).

Despite the fact that vancomycin resistance determinants are harbored by the majority of strains and sequence types of clade A1 strains (Fig. 2), ARDs linked to two important last-line antibiotics (such as linezolid and daptomycin) against VREfm infections still seem to be limited to a small percentage of clade A1 strains that belong to different sequence types. Most strains with ARDs for linezolid (*optrA*, *poxtA*, and *cfrD*) belonged to ST132 and, to a lesser extent, ST612 (Table S2). On the other hand, most strains with mutations in the *liaFSR* operon associated with the development of nonsusceptibility to DAP belong to ST736 and ST664 (Table S2).

The use of WGS-based approaches such as coreSNP (Fig. 3), cgMLST (Fig. 4), and phylogenetic analysis (Fig. 5; Fig. S2 to S6) in combination with epidemiological data from patients allowed us to identify a total of four E. faecium clonal clusters that simultaneously occurred at UAMS during the 1-year sample collection period. Isolates from these four clusters belonged to three different STs, which are among the previously identified multidrug-resistant clade A1 sequence types ST80, ST412, and ST736. The profiles of ARDs were consistent among isolates of the same clonal cluster. Most of the findings described in this study strongly suggest the existence of nosocomial transmission of the E. faecium clones within and between hospital wards; however, we also found highly genetically related clones that persisted over time and were isolated from urine samples from immunocompromised patients who came to the hospital for routine visits (Tables S4, S5, and S7). Analysis of the epidemiological data and the history of the patients demonstrated that some of the isolates belonging to the four clonal clusters were obtained from patients who routinely visit the infusion center and the emergency department, implying that these patients could have been infected from dry inanimate surfaces or from contact with other patients in common areas, such as the emergency department or the infusion center, several months before the time of sample collection. Long-term persistence of multidrug-resistant enterococcus clones in the gut of patients affected by systemic antimicrobial treatments has been documented (70, 71), as well as the existence of symptomatic and asymptomatic recurrent urinary tract infections caused by other species, such as Escherichia coli (72, 73). Thus, the antimicrobial therapy which patients with chronic and hemato-oncological diseases are subjected to, as was the case for the patients in this study, may act as a selective and driving factor that promotes persistent enterococcal colonization through the inhibition of the autochthonous flora (71) and also through the induction of the expression of factors, other than ARDs, that could promote adherence to the intestinal epithelial lining and biofilm formation (70, 74). In the case of the UAMS data set, several isolates from urine samples belonging to clonal clusters of the outbreak described in our analysis were flagged as community-acquired isolates because they were identified in a routine analysis of a nonhospitalized patient, which might prevent early infection control management in these cases. These isolates were obtained from patients with a history of previous hospital admissions, highlighting the importance of performing genomic surveillance and interventions, especially for patients at high risk, such as those with hematologic malignancies. The low infection and high colonization rates observed during this outbreak could also be explained by the low level of virulence observed in the isolates collected for this study (Table S6) (55, 75, 76).

In conclusion, in this study we performed a retrospective investigation of E. faecium isolates detected in blood and urine samples from high-risk patients who test positive for E. faecium using WGS analytical techniques in combination with clinical and epidemiological data in order to assess the genomic diversity of E. faecium isolates in the context of the species. Our findings demonstrate that the top-down genomic surveillance approach utilized in this study could accurately resolve a polyclonal outbreak of VREfm at UAMS. The addition of epidemiological data for high-risk patients who test positive for E. faecium along with ongoing genomic surveillance and intervention may be key to predicting the carriage of persistent E. faecium clones, allowing early activation of infection control measures such as screening and contact precautions among such patients.

## MATERIALS AND METHODS

### Study design.

The present study involved adult patients, with ages ranging from 18 to 95 years (inclusive), undergoing treatment at the University of Arkansas for Medical Sciences (UAMS) (Arkansas, USA) who developed a clinical infection (bloodstream or urinary tract infection as determined by blood or urine culture) with Enterococcus faecium. Patients who had blood or urine cultures positive for E. faecium (by standard clinical lab testing) were included in the study. Deidentified clinical and epidemiologic data were collected for the study participants. Clinical data includes outcome data related to hospital length of stay, length of stay in the intensive care unit (ICU), hospital unit, duration of bacteremia, antimicrobial administered, 30- and 9-day mortality, and development of graft-versus-host disease. The study was approved by the Institutional Review Board of UAMS (IRB no. 228137).

### Bacterial isolates.

A total of 106 E. faecium isolates were identified from positive blood and urine cultures from 66 patients. Samples collected were grown on blood cultures and processed on the BacT/Alert 3D (bioMérieux) system. Positive blood cultures were then subcultured on blood agar plates. Isolated colonies were used for identification and susceptibility testing using the Vitek MS and Vitek 2 systems.

### DNA extraction and quantification.

Microbial DNA was extracted from pure growth of VREfm. Isolated colonies on the blood agar plates were picked and resuspended in a DNA/RNA Shield collection and lysis tube (Zymo Research, Irvine, CA). Genomic DNA was extracted from the tube using a Quick-DNA fungal/bacterial kit (Zymo Research, Irvine, CA). The purity of extracted DNA was determined using a NanoDrop spectrophotometer by measuring the A260/A280 and A260/A230 ratios. DNA integrity and quantity were determined using an Agilent 2200 TapeStation and Qubit 3.0 assay, respectively.

### Vancomycin and daptomycin susceptibility testing.

Vancomycin resistance was confirmed using E-tests (bioMérieux). Urine cultures were processed similarly using blood agar plates for isolation of colonies. Antimicrobial susceptibility test results were interpreted using the M100 CLSI standards (77).

### Illumina sequencing and genome assembly.

Paired-end 150-bp libraries were constructed using the KAPA HyperPlus kit (Roche) with enzymatic fragmentation for 10 min. The resulting genomic libraries of the E. faecium isolates were sequenced using the Illumina NextSeq 550 platform at the UAMS Myeloma Center. Adapters were trimmed using fastp v0.19.5 (78) with default settings. Trimmomatic v0.38 (79) was used to remove poor-quality reads, with the following parameters: HEADCROP:15 LEADING:20 TRAILING:20 SLIDINGWINDOW:5:20 MINLEN:50. The quality of pre- and postprocessed reads was assessed with FastQC v0.11.8 (80). The resulting high-quality reads were assembled *de novo* using SPAdes v3.13.0 (81) with the settings, “error-correction” and “careful,” k-mer sizes of 21, 33, 55, and 77, and a minimum contig size of 500 bp. Draft genomes were quality checked using default settings in QUAST v5.0.2 (82). Genome sequences were submitted for annotation to the NCBI Prokaryotic Genome Annotation Pipeline (PGAP) (83) using the default parameters.

### MLST and cgMLST scheme creation.

MLST classification of the 1,384 clade A1 strains was done using mlst software from T. Seemann (https://github.com/tseemann/mlst), which incorporates components of the PubMLST database (https://pubmlst.org/) developed by Keith Jolley (84) and sited at the University of Oxford. cgMLST classification of the set of 106 UAMS E. faecium isolates was obtained using chewBBACA suite v2.0.16 (85). The minimum spanning tree was plotted using PHYLOVIZ v2.0 applying goeBURST clustering algorithm (86).

### coreSNP analysis.

Mapping of SNP calling analyses was done using Snippy v4.6 (https://github.com/tseemann/snippy). Pairwise SNPs were calculated in R using harrietr (v0.2.3; https://github.com/andersgs/harrietr) and coreSNP alignments from Snippy without masking prophage or recombination regions, as is considered best for accurate and consistent multidrug-resistant-organism transmission inference when using core genome alignments and SNP thresholds (58, 66). The data from the coreSNP analysis are presented as a clustered heat map using R and the ComplexHeat map v2.12.1 library (87).

### FastANI tree.

ANI measurements were obtained using FastANI v1.33 software (29) with a k-mer length of 21 and were used to construct a distance matrix (100 - ANI similarity measure). Distance-based trees were built using ape R package (88) and ward.D2 clustering algorithm. Trees were visualized using the Interactive Tree of Life (iToL) v6.2 (89).

### Antibiotic resistance determinant and virulence factor annotation.

Antibiotic resistance determinants were predicted using the Resistance Gene Identifier (RGI) v5.2.0 against the Comprehensive Antibiotic Resistance Database (CARD) v3.1.1 (47), and virulence factors were identified using the ABRicate v1.0.0 screening tool using a custom database of virulence factors downloaded from NCBI nucleotide database and the Virulence Factor Database (VFDB), accessed on 26 August 2022 (90, 91). Virulence factors with more than 80% sequence coverage and sequence identity were annotated in the assemblies of clade A1 strains.

### Phylogenetic tree.

Alleles obtained from the cgMLST analysis and present in all strains (1,266 alleles) were extracted and individually aligned using MAFF v7.475 (92) with the options –maxiterate 1000 –globalpair and using the algorithm G-INS-i for the alignment strategy, which assumes that entire regions can be aligned and tries to align them globally using the Needleman-Wunsch algorithm. Alignments were trimmed using ClipKIT v1.30 (93) and concatenated to be used as input for IQ-TREE v2.2.0.3 to construct a phylogenetic tree using a maximum-likelihood approach (94). ModelFinder (95) was used to find the best model for each partition in IQ-TREE (-m MFP+MERGE). For the tree reconstruction, 1,000 ultrafast bootstraps (-B 1000) (96) were used to evaluate the nodal support. The tree was rooted using clade A2 isolates.

### Data availability.

Raw sequencing data, assembly, and functional annotations for the isolates used in this study are available under the BioProject accession numbers PRJNA518133, PRJNA735268, and PRJNA520878 in the NCBI database.
