# Supplementary material for: Top-Down Genomic Surveillance Approach To Investigate the Genomic Epidemiology and Antibiotic Resistance Patterns of Enterococcus faecium Detected in Cancer Patients in Arkansas
Source: Microbiol Spectr. 2023 Mar 30;11(3):e04901-22. doi: 10.1128/spectrum.04901-22 (PMC10269635; doi:10.1128/spectrum.04901-22)
Supplement: Supplemental file 8 — Fig. S1 to S7. Download spectrum.04901-22-s0001.pdf, PDF file, 2.3 MB [file spectrum.04901-22-s0001.pdf]

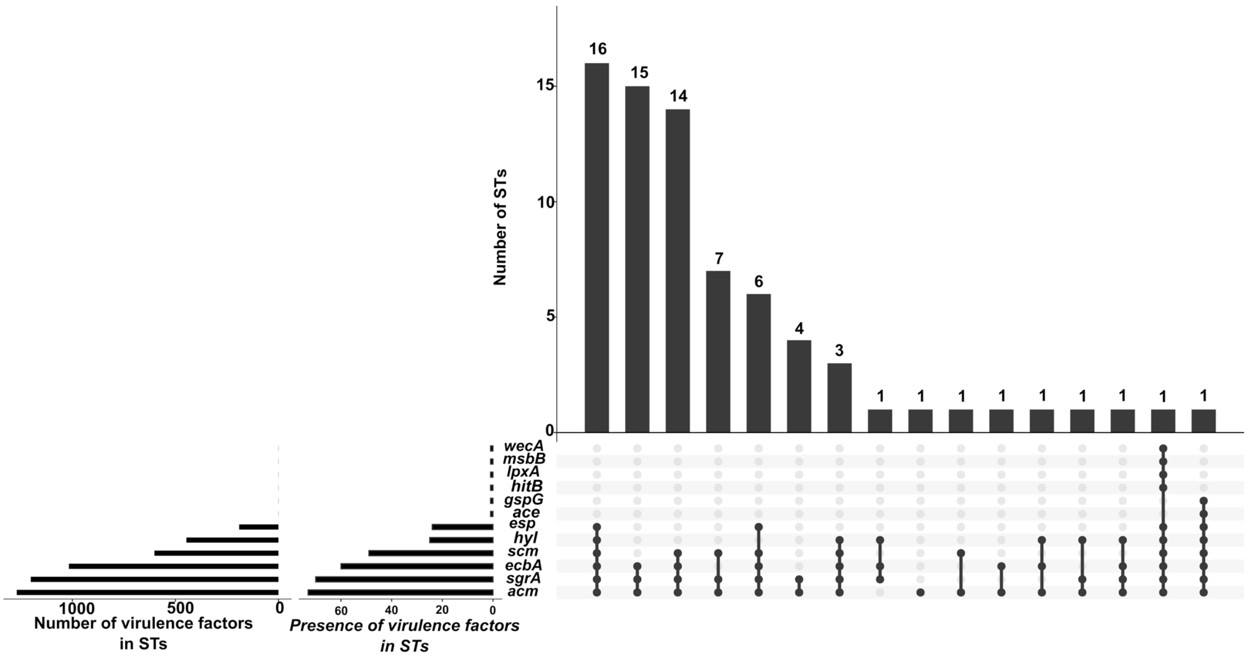

Suppl. Figure 1. MLST and virulence factor intersection plot. Numbers above the bars represent the number of sequence types for each set of virulence factors (indicated by black circles in the intersection plot).

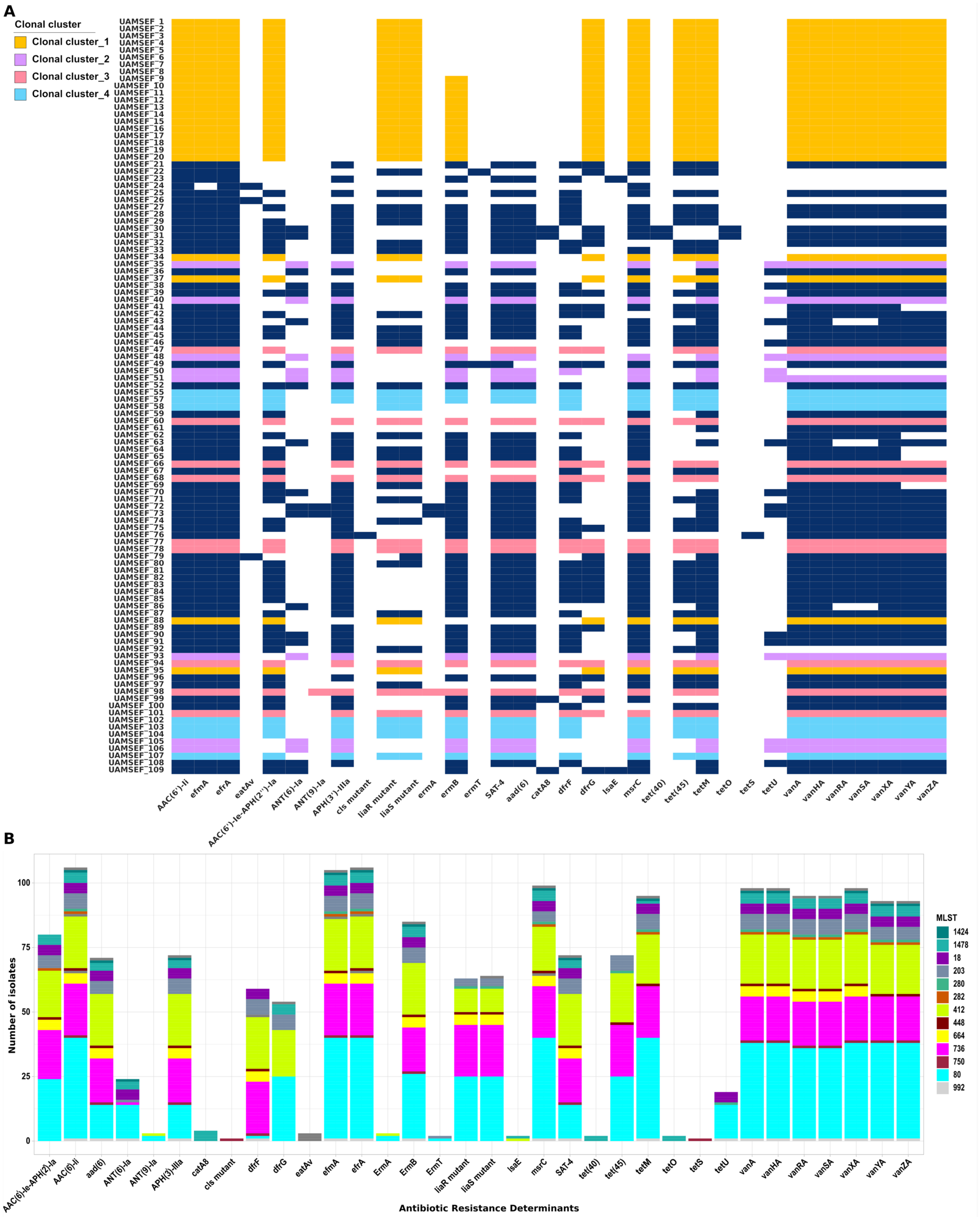

Suppl. Figure 2. A. Antibiotic resistance patterns of 106 isolates collected from cancer patients at UAMS. Blue color blocks represent ARDs from isolates that do not belong to clonal clusters. **B.**

Stacked barplot of ARDs identified in 106 isolates collected from cancer patients at UAMS with MLST distribution. A total of 34 non-redundant ARDs and 13 sequence types, were identified in the 106 isolates.

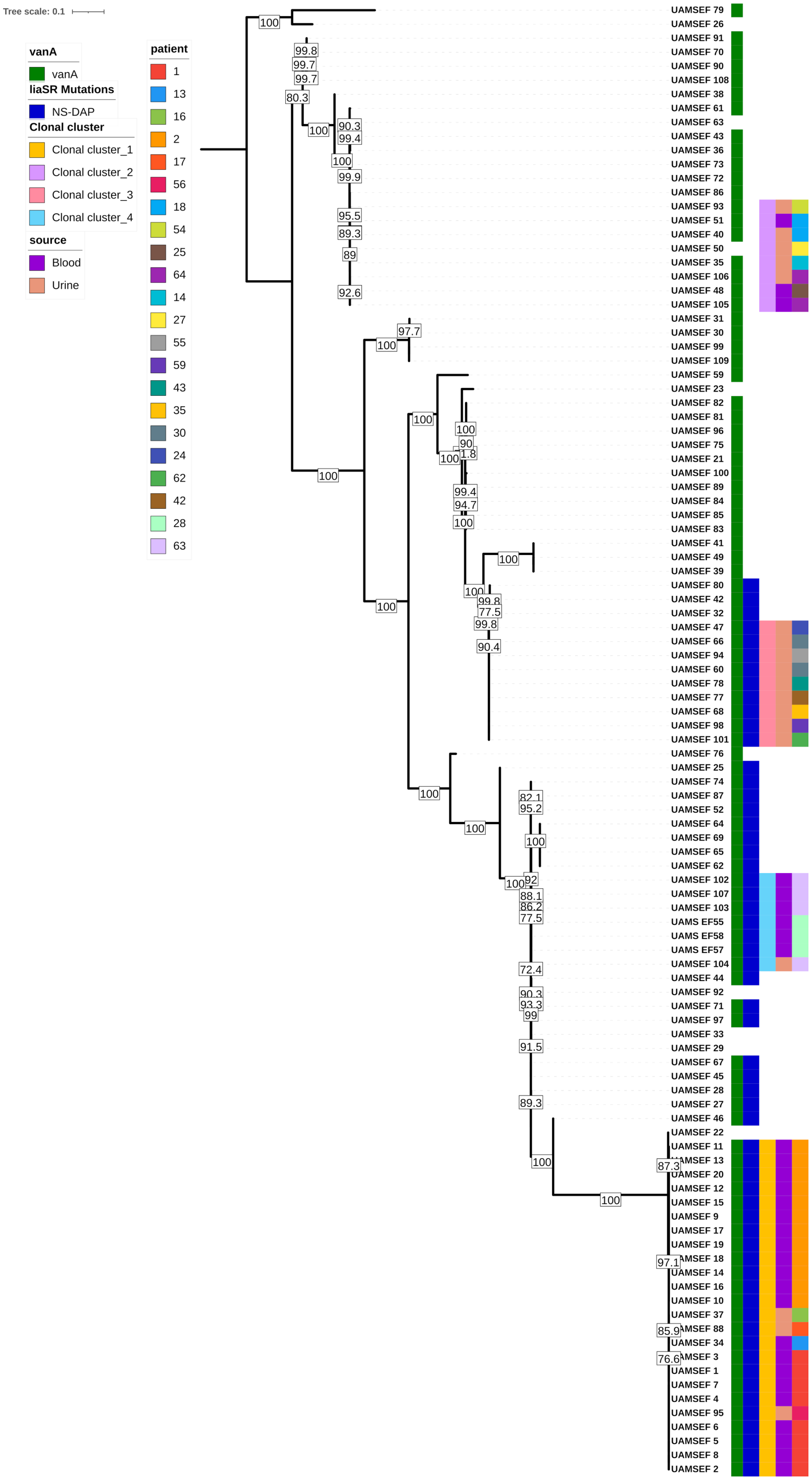

Suppl. Figure 3. Maximum likelihood phylogenetic tree of 105 clade A1 and A2 *E. faecium*

isolates. Isolates belonging to each of the four detected clonal clusters are marked in the inner ring of the tree. Presence of *vanA* cluster of genes conferring resistance to vancomycin and mutations in *liaSR* genes related to daptomycin non-susceptible phenotype are represented in the second and third bar respectively. Bootstrap values are represented in the tree branches.

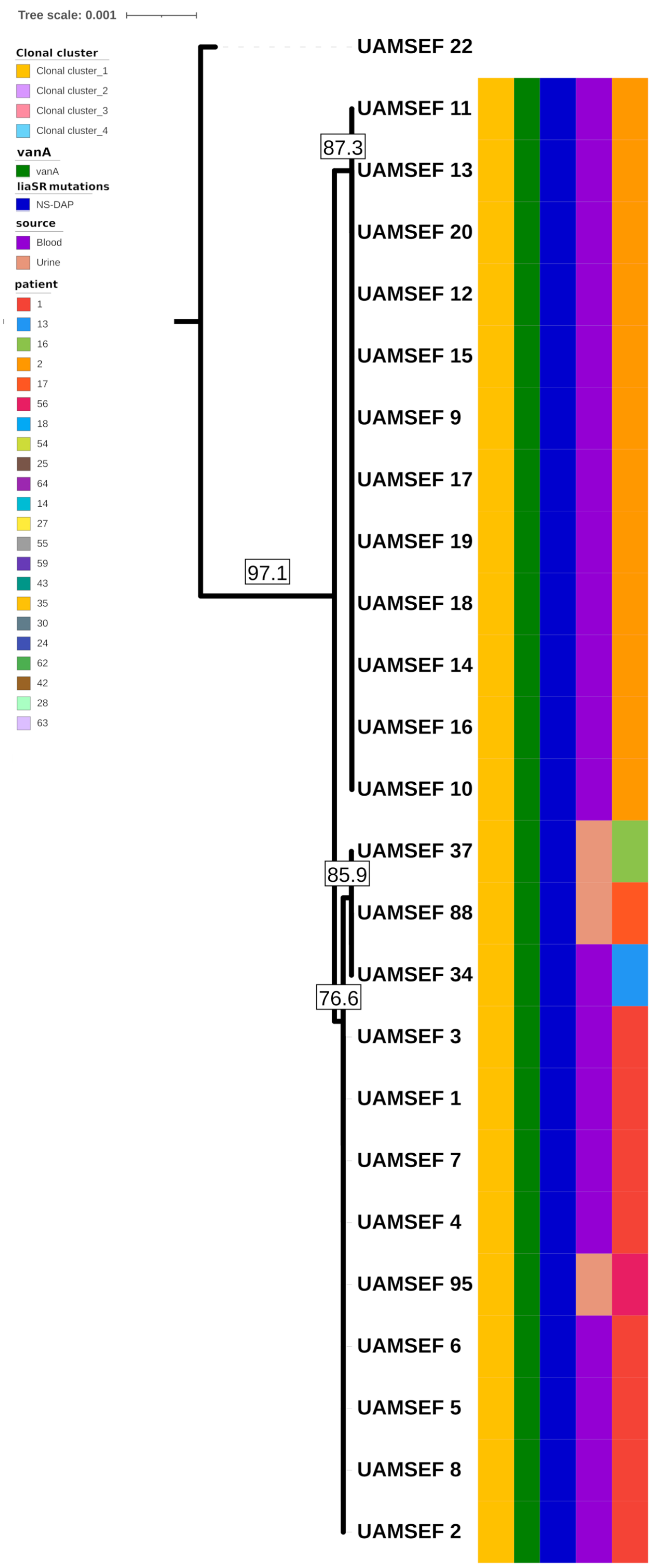

Suppl. Figure 4. Pruned maximum likelihood phylogenetic tree of clade A1 and A2 *E. faecium* isolates which contains strains from clonal cluster 1. Presence of *vanA* cluster of genes conferring

resistance to vancomycin and mutations in *liaSR* genes related to daptomycin non-susceptible phenotype are represented in the second and third bar respectively. Bootstrap values are represented in the tree branches.

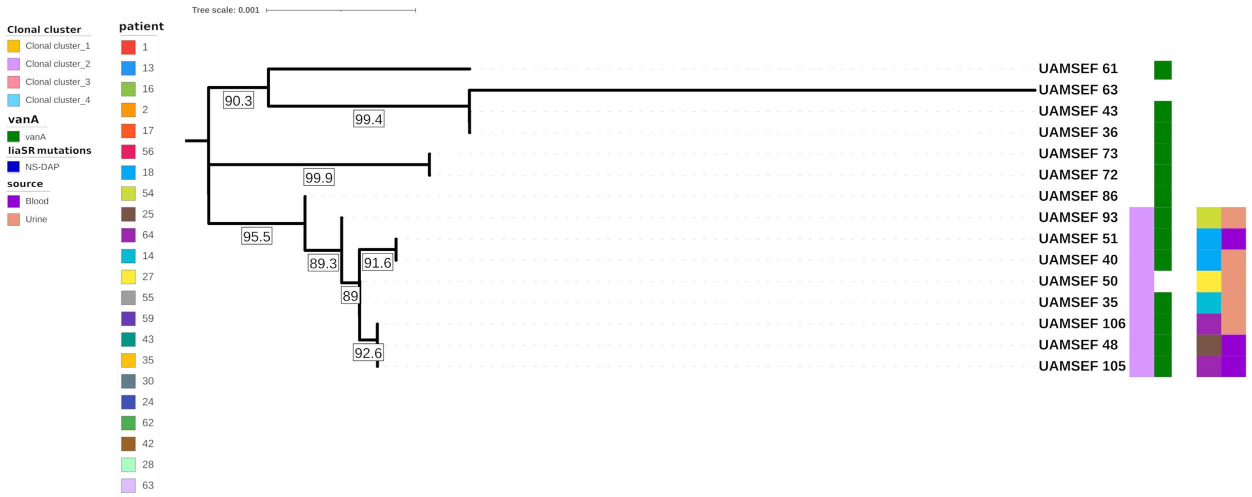

Suppl. Figure 5. Pruned maximum likelihood phylogenetic tree of clade A1 and A2 *E. faecium* isolates which contains isolates from clonal cluster 2. Presence of *vanA* cluster of genes conferring resistance to vancomycin and mutations in *liaSR* genes related to daptomycin non-susceptible phenotype are represented in the second and third bar respectively. Bootstrap values are represented in the tree branches.

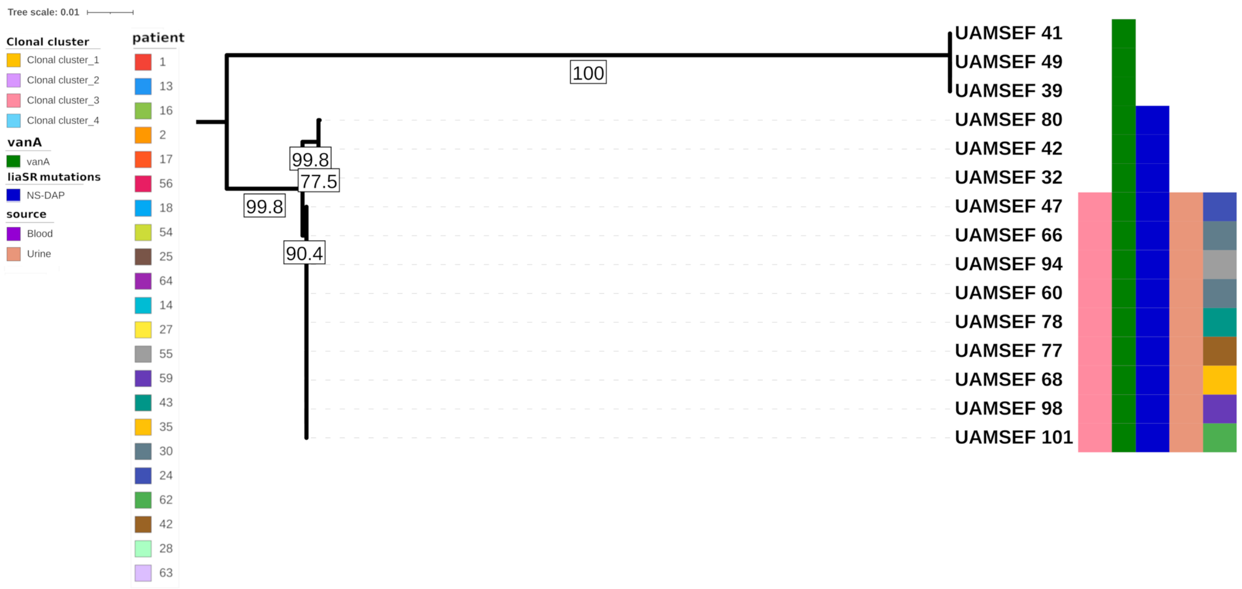

Suppl. Figure 6. Pruned maximum likelihood phylogenetic tree of clade A1 and A2 *E. faecium* isolates which contains isolates from clonal cluster 3. Presence of *vanA* cluster of genes conferring resistance to vancomycin and mutations in *liaSR* genes related to daptomycin non-susceptible phenotype are represented in the second and third bar respectively. Bootstrap values are represented in the tree branches.

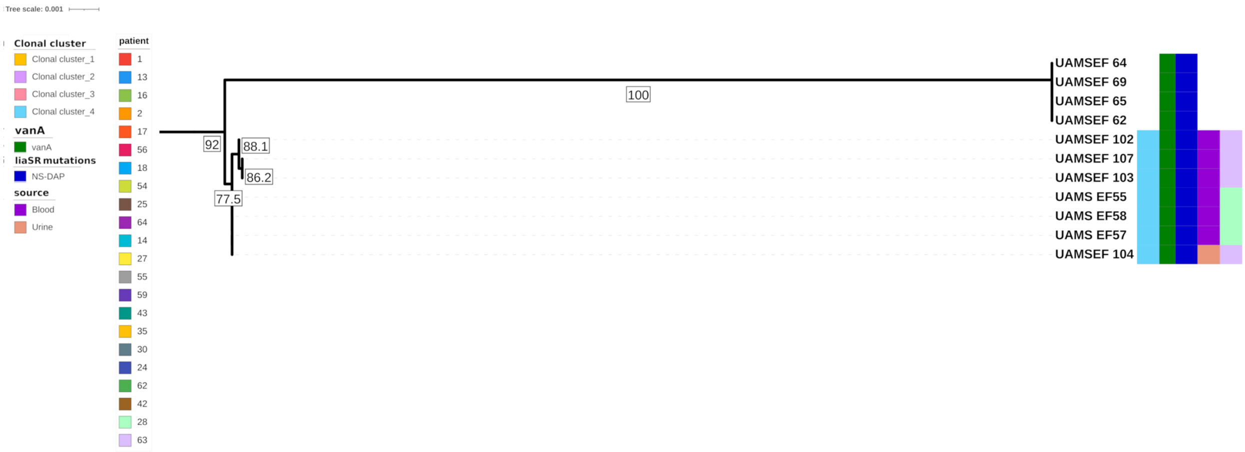

Suppl. Figure 7. Pruned maximum likelihood phylogenetic tree of clade A1 and A2 *E. faecium* isolates which contains isolates from clonal cluster 4. Presence of *vanA* cluster of genes conferring resistance to vancomycin and mutations in *liaSR* genes related to daptomycin non-susceptible

phenotype are represented in the second and third bar respectively. Bootstrap values are represented in the tree branches.
